# Identifying the most recommended novel teaching strategy in orthopaedics education: a systematic review and network meta-analysis

**DOI:** 10.3389/fmed.2026.1762807

**Published:** 2026-02-10

**Authors:** Hongxin Cao, Zhongjie Ji, Linyang Song, Hongliang Wang, Yunzhen Chen, Guangjun Jiao

**Affiliations:** 1 Department of Medical Oncology, Qilu Hospital of Shandong University, Jinan, Shandong, China; 2 Department of Spine Surgery, Qilu Hospital of Shandong University, Jinan, Shandong, China

**Keywords:** network meta-analysis, orthopaedics education, problem-based learning, teaching strategies, team based learning (TBL), VR

## Abstract

**Background:**

Despite the growing adoption of novel teaching strategies in orthopaedics education, their comparative effectiveness remains unclear. This network meta-analysis (NMA) evaluates and ranks the efficacy of problem-based learning (PBL), virtual reality (VR), Video, three dimensions (3D) simulations, flipped classrooms (FC), 3D combined PBL, FC combined team based learning (TBL), and traditional lecture-based learning (LBL) in orthopaedic education.

**Methods:**

A systematic search of PubMed, Web of Science, Embase, and Cochrane Library was conducted up to December 31, 2024. Randomized controlled trials (RCTs) comparing teaching strategies in orthopaedics education were included. Specific criteria were utilized to identify relevant studies, and data extraction was subsequently carried out. Outcomes included theoretical knowledge, procedural or clinical skills, and learner satisfaction. Pairwise and network meta-analyses were performed using R software.

**Results:**

After screening 893 studies, 11 RCTs involving 690 medical students or residents were included in the NMA. VR was more effective than LBL for procedural or clinical skills (SMD = 6.88, 95% CI: 1.05–12.13), while FC + TBL improved theoretical test scores with the highest SUCRA probability (81.73%). FC + TBL also enhanced student satisfaction (SMD = 1.42, 95% CI: 0.04–2.79), with PBL having the highest SUCRA probability (61.53%) for this outcome.

**Conclusion:**

Our NMA found FC + TBL and VR to be the most effective novel teaching strategies in orthopaedics education for improving theoretical and clinical skill scores, respectively. However, differences among strategies were minor. Future studies with larger samples, diverse populations, and more outcome measures are needed for a comprehensive evaluation.

## Background

Orthopedics, a field deeply rooted in anatomical mastery, biomechanical logic, and dynamic surgical decision-making, faces persistent challenges in fostering well-rounded competencies. Traditional lecture-based learning (LBL), which emphasizes rote memorization of concepts like fracture classifications or implant protocols, struggles to bridge the gap between theory and real-world application. Studies have revealed that under LBL, orthopedic residents exhibit a 31% error rate in biomechanical analysis of complex cases and significantly lag in managing postoperative complications compared to active learning approaches (1). In China’s current system, the fourth year of medical school focuses on theory, while the fifth shifts to clinical practice, yet both phases rely heavily on standardized curricula. This rigid structure stifles critical thinking and teamwork skills—72% of trainees report limited exposure to real surgical challenges during internships, as lessons remain textbook-bound (2). This “theory-practice divide” contributes to burnout (affecting 18% of students) and skill gaps: only 43% of graduates from top Chinese medical schools meet competency standards in emerging technologies like robot-assisted surgery (3), far below global benchmarks. Thus, reforming orthopedic education to integrate active learning, dynamic feedback, and adaptability to new technologies has become urgent.

To address these limitations, innovative strategies like virtual reality (VR), 3D interactive systems, and flipped classrooms (FC) are transforming training. VR simulations replicate real surgical environments, boosting skill transfer efficiency. Vallée et al. (1) found VR-trained residents completed arthroscopic procedures 42% faster, with 14% higher accuracy in tissue identification than traditional apprenticeship trainees. Xue et al. (3) developed a 3D Training System that integrates real imaging with live anatomical guides, tripling femoral neck fracture classification accuracy while reducing cognitive load by 28%. Meanwhile, FC combined with team-based learning (TBL) redefines roles: pre-class self-study, in-case debates, and post-surgery debriefs raised clinical decision-making scores by 9.2% and complication prediction accuracy to 72.3% (4). A study of 130 students found that podcast/videos users significantly outperformed text users in posttests and knowledge gain, with higher approval ratings for podcasts/videos (5). Recent quasi-experimental work further supports the efficacy of integrated digital approaches; for instance, massive open online course (MOOC)–virtual simulation combinations have demonstrated significant improvements in surgical skill acquisition, including wound debridement and basic operative techniques (6, 7). Beyond building muscle memory for procedures, these tools provide visual data and instant feedback to correct errors—opportunities missing in traditional teaching.

Despite their clear benefits, debates persist regarding the best strategies. A key issue is the lack of direct comparisons between methods. Moreover, conflicting conclusions across meta-analyses could limit the scalable implementation of innovative teaching methods in educational and clinical environments (8–11). Despite the suggestion that specific teaching environments should undergo thorough evaluation when introducing new teaching strategies (12), there remains a dearth of research to substantiate this notion. Here, systematic reviews and network meta-analysis (NMA)—considered the highest level of evidence—can help by comparing multiple methods at once. In this study, we employ NMA integrated with statistical ranking to systematically evaluate eight mainstream educational approaches (LBL, problem-based learning (PBL), video, VR, 3D, 3D + PBL, FC and FC + TBL) in fostering theoretical knowledge assimilation and clinical skill proficiency. The findings aim to establish a data-driven decision-making framework for optimizing resource allocation across diverse educational and clinical contexts.

## Methods

The systematic review and NMA adhered to the Preferred Reporting Items for Systematic Reviews and Meta-Analyses (PRISMA) extension statement (13), with the objective of assessing and comparing the efficacy of eight individual teaching strategies in enhancing orthopedic education by examining three specific indicators (14). This review did not require ethical approval as it utilized data from published studies, and no detailed participant information was made public.

### Literature retrieval strategy

Two authors (Jiao G and Cao H) conducted a thorough literature review by employing both database searches and manual search methods. The electronic databases listed below were searched through December 2024: PubMed, Web of Science, Embase, and Cochrane library. The search strategies, designed for reproducibility, were conducted based on the Population, Intervention, Comparison, and Outcome (PICO) framework, as detailed in Supplementary Appendix 1. Moreover, pertinent randomized controlled trials (RCTs) were located through manual searches of the reference lists in relevant meta-analyses and prominent medical education journals. In cases of disagreement between the two pairs of authors (e.g., during literature screening, data extraction, or quality assessment), the discrepancies were first resolved through joint discussion. If consensus could not be reached, a third independent senior researcher (Chen Y) with expertise in orthopaedic education and systematic reviews was consulted to provide an objective judgment. The final decision was made based on the third researcher’s recommendations, and all resolution processes were documented in detail to ensure transparency and minimize bias.

### Inclusion and exclusion criteria

#### Inclusion criteria

Two authors (Ji Z and Song L) independently screened all retrieved studies using Zotero (version 7.0.11) literature management software, developed by the Corporation for Digital Scholarship, USA, based on predetermined inclusion and exclusion criteria. Studies that satisfied the following inclusion criteria were included: (1) participants were medical students, interns, or resident doctors, without regard to gender, age, grade, ethnicity, nationality, or educational background; (2) the focus was on orthopedic-related education; (3) comparisons were made between eight novel teaching methods and LBL method; (4) outcomes were assessed using: knowledge scores to gauge theoretical understanding; procedural skill scores for operational skills like fracture reduction and trauma management; clinical skill scores for practical clinical problem-solving abilities, including history taking, examination, diagnosis, and treatment planning; total scores combining the above to evaluate overall abilities; and questionnaire surveys to assess teaching methods, including interest, satisfaction, problem-solving ability, learning time/pressure, independence, teamwork, communication, and clinical reasoning.; (5) RCTs were included; and (6) the publications were in English.

#### Exclusion criteria

Studies were excluded if: (1) full-text data were unavailable after attempts to retrieve from authors or institutional repositories; (2) no quantifiable outcome measures (e.g., knowledge scores, skill performance, satisfaction ratings) were reported; (3) they were retracted, or published as abstracts only.

#### Data extraction

Two independent reviewers (Cao H and Song L) extracted data from the included studies, adhering to the guidelines set forth by the Cochrane Collaboration for Systematic Reviews. They each independently reviewed the full texts of studies that potentially met the inclusion and exclusion criteria, and subsequently extracted the relevant data as followed: name of the first author, year of publication, participants characteristics, number of participants, intervention, comparison, study duration, outcome assessment measures and study design type.

#### Quality assessment

Utilizing the Cochrane Collaboration’s Risk of Bias 2.0 tool (RoB2) (15), two independent investigators (Ji Z and Song L) systematically evaluated six following biases of the included articles: (1) bias arising from the randomization process; (2) bias due to deviations from intended interventions; (3) bias due to missing outcome data; (4) bias in the measurement of the outcome; (5) bias in selection of the reported result and (6) overall bias (16). Each item was classified as high risk, low risk or some concern.

### Statistical analyses

#### Pairwise meta-analysis

The pairwise meta-analyses were performed using a random-effects model in R 4.3.3 software (R Core Team) with the “meta,” “netmeta,” “gemtc,” and “ggplot2” packages to examine the direct evidence. Because all outcome measures were continuous, we opted to use standardized mean differences (SMDs) as the measure of effect size, along with 95% confidence intervals (CIs), to account for the variety of rating scales employed in the studies included (17). To assess statistical heterogeneity in each pairwise comparison, we utilized the *p*-value of the Q-test, the *I*^2^ statistic, and the between-study variance (τ^2^).

#### Network meta-analysis

This NMA was conducted to assess and rank seven innovative teaching strategies in orthopaedics education by integrating both direct and indirect comparative analyses. The three outcome measures were presented as SMDs with accompanying 95% CIs, and each was analyzed separately. To ensure more cautious conclusions, irrespective of heterogeneity, a random-effects model was adopted (18). The network plots were created using R 4.3.3 software. Furthermore, league tables and forest plots were constructed to showcase the effectiveness of all pairwise comparisons of teaching strategies in terms of effect sizes. The surface under the cumulative ranking curve (SUCRA) was used to rank the relative effectiveness of the teaching strategies. SUCRA is a key metric in NMA that quantifies the probability of an intervention (here, a teaching strategy) being the best, second-best, or worst among all compared options. It ranges from 0 to 100%, where a higher SUCRA value indicates a greater likelihood of being the most effective strategy. Unlike traditional pairwise meta-analysis, SUCRA integrates both direct and indirect comparative evidence from the network, providing a comprehensive and intuitive ranking of multiple interventions. We used SUCRA because it addresses the limitation of pairwise comparisons by synthesizing all available evidence, making it particularly valuable for our study—where we compared eight distinct teaching strategies—to identify the most promising approaches for orthopaedic education. A bar graph was also used to visually depict the SUCRA probabilities for each teaching strategy, aiding in a comparative examination of their influence on the three outcome indicators. To identify any discrepancies between direct and indirect comparisons, the node-splitting approach was utilized (19, 20).

## Results

### Characteristics of included studies

**Figure 1:**
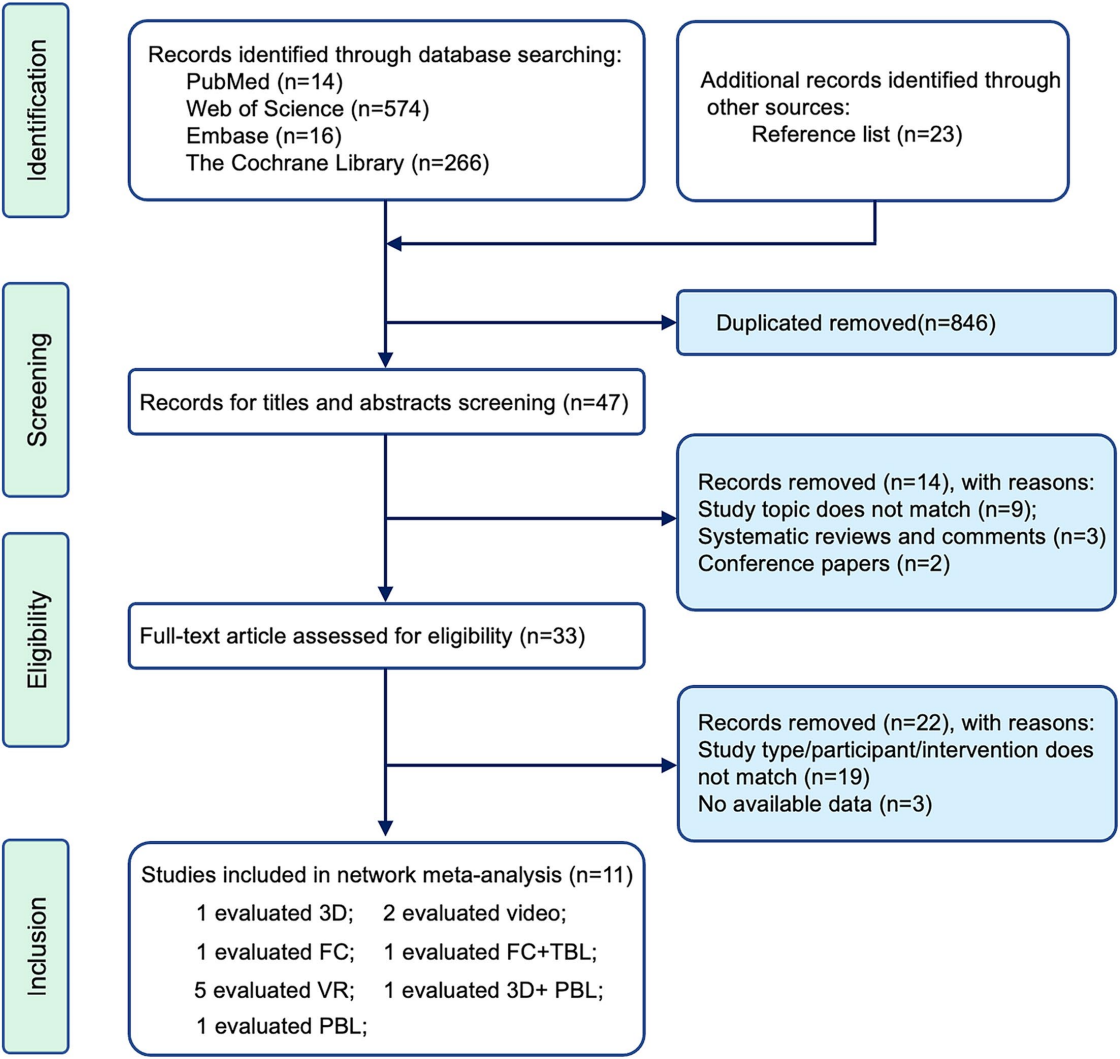
The flowchart of the study.

After conducting thorough searches and removing duplicates, we identified 893 potential studies for further evaluation. These studies were then screened for eligibility based on their titles and abstracts, leading to the exclusion of 860 studies, leaving 33 for further consideration. Following a full-text assessment, 22 articles were excluded for various reasons. Ultimately, 11 articles (1–5, 21–26) met the predefined inclusion and exclusion criteria and were chosen for inclusion in the NMA, as illustrated in Figure 1.

Table 1 presents an overview of the key features of the 11 RCTs involving 690 medical education students or residents. Among these studies, three reported that 54.20% (374 out of 690) of the participants were undergraduate students. The outcome measures differed across the trials: 7 concentrated on theoretical exam scores, 7 on practical or experimental exam scores, and 4 assessed student satisfaction levels.

**Table 1:** Basic characteristics of the included literature.

Study ID	Nation	Participants	Intervention	Control	Number (Interv./Ctl)	Outcome measurements	Teaching subjects
Xue et al. (2024) (3)	China	Resident	3D	LBL	30/30	① ②	Proximal humerus fractures
Xue et al. (2024) (3)	China	Resident	Video	LBL	29/30	① ②	Proximal humerus fractures
Xue et al. (2024) (3)	China	Resident	3D	Video	30/29	① ②	Proximal humerus fractures
Wang et al. (2024) (2)	China	Undergraduate	FC	LBL	69/69	① ②③	Basic theory
Vallée et al. (2024) (1)	France	Orthopaedic residents	VR	LBL	13/13	②	Rotator cuff repair
Capitani et al. (2024) (21)	Italy	Orthopaedic residents	VR	LBL	3/3	①	Kyphoplasty
Shuai et al. (2023) (4)	China	Clinical internship students	FC + TBL	LBL	55/54	① ③	Basic theory
Sun et al. (2022) (22)	China	5-year undergraduate students	3D + PBL	LBL	53/53	① ③	Spinal anatomy, basic steps of common spinal surgery
Lohre et al. (2020) (23)	Canada	Orthopaedic residents	VR	LBL	9/9	②	Reverse shoulder arthroplasty
Logishetty et al. (2019) (24)	United Kingdom	Orthopaedic residents	VR	LBL	12/12	②	Total hip arthroplasty
Hooper et al. (2019) (25)	United States	Orthopaedic residents	VR	LBL	7/7	②	Total hip arthroplasty
Cong et al. (2017) (26)	China	Orthopaedic residents	PBL	LBL	15/15	① ②③	Spine surgical skills
Back et al. (2017) (5)	Germany	Medical students	Video	LBL	75/55	①	Basic theory

① Theoretical test scores; ② procedural or clinical skill scores; ③ students’ satisfaction scores. PBL, problem-based learning; VR, virtual reality; 3D, three dimensions; FC, flipped classrooms; TBL, team-based learning; LBL, lecture-based learning; FC + TBL, flipped classrooms combined with team-based learning.

### Quality of included studies

**Figure 2:**
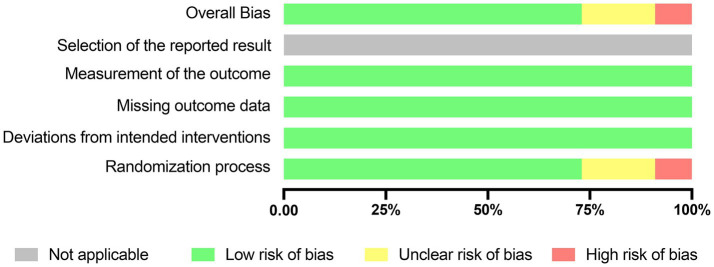
Risk assessment of bias using the RoB2. Risk of bias items of all included studies are indicated as the percentages.

Using the RoB2 tool for quality evaluation, Figure 2 presents an overview of the results assessed from the 11 RCTs included. Among these studies, one study (9%) was determined to have a high risk of bias, two (18%) raised some concerns, and eight (73%) were considered to have a low risk of bias.

When assessing the randomization process, it was found that 8 studies (73%) were deemed to have a low risk of bias due to the proper use of random sequence generation methods. Two article (18%) raised some concerns in this regard. Furthermore, all studies reported outcomes for every participant, leading to a low risk of bias assessment concerning missing outcome data in all cases (Supplementary Appendix 2). Given that preregistration and protocols are not mandatory in medical education research trials (27), the risk of bias related to selective reporting of results is not applicable in this context.

### Heterogeneity assessment

We conducted a heterogeneity analysis for each variable and observed significant heterogeneity (*I*^2^ ≥ 50%) in the comparison of Video versus LBL (77.6%) in theoretical test scores and VR versus LBL (67.9%) in procedural or clinical skill scores (Supplementary Appendix 4A,B). Regarding the students’ satisfaction score, since only a single study was included for each teaching method, heterogeneity test could not be performed. Consequently, the certainty of evidence for this outcome is very low. Future research should employ standardized, psychometrically validated tools to enable meaningful cross-intervention comparisons.

### Pairwise meta-analyses

When it comes to the impact of theoretical test results, seven innovative teaching methods—namely 3D, video, VR, FC, FC combined with TBL, PBL, and PBL integrated with 3D—did not show any significant advantage over the traditional LBL approach (Supplementary Appendix 3A). However, in terms of procedural or clinical skill assessments, VR emerged as a more effective method compared to LBL (SMD = 6.88, 95% CI: 1.05–12.13; Supplementary Appendix 3B). Additionally, student satisfaction ratings indicated that FC paired with TBL was more effective than LBL (SMD = 1.42, 95% CI: 0.04–2.79; Supplementary Appendix 3C).

### Network meta-analyses

#### The theoretical test scores

**Figure 3:**
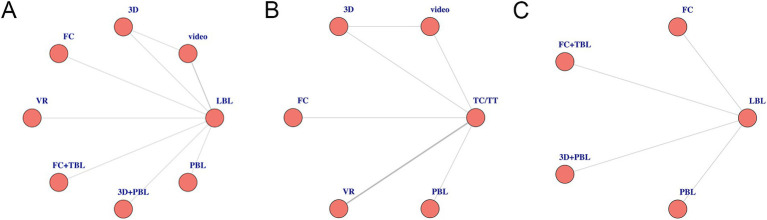
A network diagram of comparable studies for each outcome in the Bayesian network meta-analysis. **(A)** Theoretical test scores; **(B)** procedural or clinical skill scores; **(C)** students’ satisfaction scores. PBL, problem-based learning; VR, virtual reality; 3D, three dimensions; FC, flipped classrooms; TBL, team-based learning; LBL, lecture-based learning; FC + TBL, flipped classrooms combined with team-based learning.

Out of the 11 studies conducted, 7 involving 608 students and residents provided data on theoretical test scores (2–5, 21, 22, 26). This NMA comprehensively assessed the impact of various teaching methods, including video (2 studies), 3D (1 study), FC (1 study), PBL (1 study), FC combined with TBL (1 study), VR (1 study), and 3D integrated with PBL (1 study). A detailed network diagram illustrating all comparisons is shown in Figure 3A.

**Figure 4:**
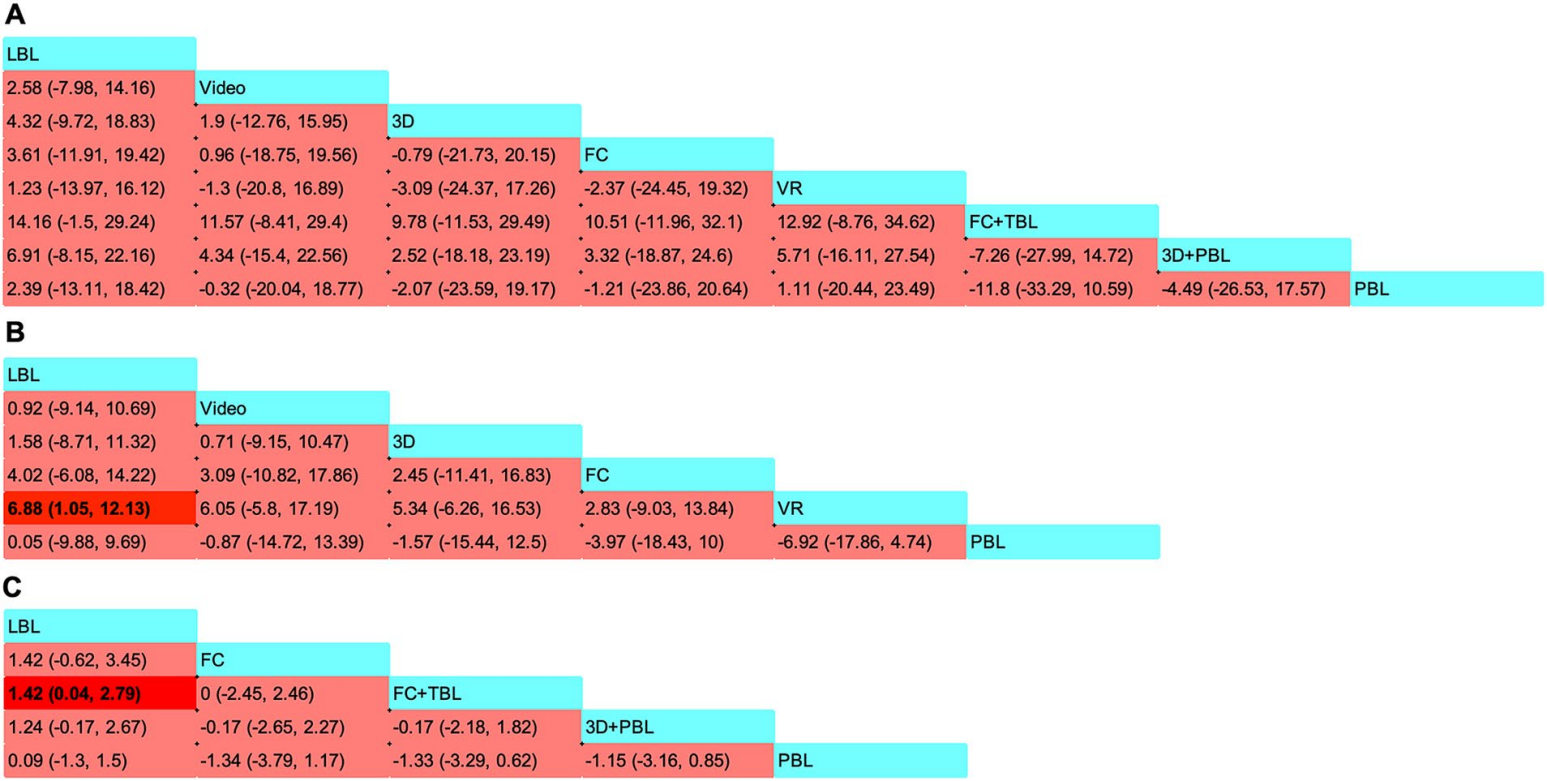
Pooled estimates of the network meta-analysis. **(A)** Standard mean differences (SMDs) (95% CI) of the theoretical test scores; **(B)** SMDs (95% CI) of the procedural or clinical skill scores; **(C)** SMDs (95% CI) of the students’ satisfaction scores. SMDs greater than zero indicate preference for the method defined by the column. Significant findings are emphasized in bold with background shading. PBL, problem-based learning; VR, virtual reality; 3D, three dimensions; FC, flipped classrooms; TBL, team-based learning; LBL, lecture-based learning; FC + TBL, flipped classrooms combined with team-based learning.

**Figure 5:**
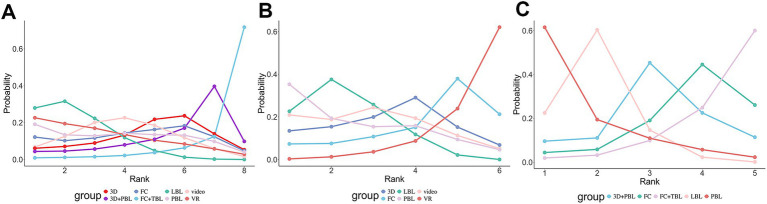
The results of Bayesian ranking for primary outcomes. The line graph presents the ranking probabilities of various treatment options from first to last in terms of theoretical test scores **(A)**, procedural or clinical skill scores **(B)**, and students’ satisfaction scores **(C)**. The abscissa represents “Rank,” and the ordinate represents “Probability.” Different intervention measures are distinguished by lines of different colors. The ranking probability for each intervention corresponds to the position of the circle on the ordinate. PBL, problem-based learning; VR, virtual reality; 3D, three dimensions; FC, flipped classrooms; TBL, team-based learning; LBL, lecture-based learning; FC + TBL, flipped classrooms combined with team-based learning.

The SMD values and 95% CI derived from NMA are shown in Figure 4A. The 95% CI for the comparisons of these teaching strategies included zero, indicating that there was no statistically significant difference among the seven innovative teaching approaches. The SUCRA rankings and probability values presented in Figure 5A indicate that FC + TBL has the highest likelihood of improving theoretical test scores in orthopedic education, with a probability of 81.73%. FC + TBL ranked first for theoretical performance; however, its estimated effect versus LBL was not statistically significant (SMD = 14.16, 95% CI: −1.5 to 29.24).

#### Procedural or clinical skill scores

Seven studies, encompassing 337 students or residents, reported on this indicator (1–3, 23–26). The NMA included various methods: video (*n* = 1), 3D (*n* = 1), FC (*n* = 1), PBL (*n* = 4), and VR (*n* = 4). The specifics of other comparisons can be found in the network diagram presented in Figure 3B.

Figure 4B showcased the precise SMD figures along with their 95% CI derived from the NMA. Students utilizing VR strategies attained markedly higher scores in procedural or clinical skill assessments when compared to those employing LBL strategies, with an SMD of 6.88 (95% CI: 1.05–12.13). It’s worth noting that no discernible differences were found in the indirect comparisons between the various novel teaching approaches. The SUCRA ranking and probability values presented in Figure 5B suggested that VR (with a probability of 62.07%) was the most probable approach to enhance scores in procedural or clinical skill tests.

#### The students’ satisfaction score

A thorough examination of four studies (2, 4, 22, 26) involving 383 students presented their satisfaction ratings, which reflect the students’ subjective assessments and perspectives on diverse teaching approaches. The teaching methods examined in this NMA included FC (*n* = 1), FC combined with TBL (*n* = 1), 3D integrated with PBL (*n* = 1), and PBL alone (*n* = 1). Figure 3C presents the network diagram illustrating all the comparisons made.

As depicted in Figure 4C, students who learned using the FC + TBL strategy exhibited significantly higher satisfaction scores compared to those who learned using LBL (1.42, with a 95% CI ranging from 0.04 to 2.79). These results consistent with the findings from the pairwise meta-analyses. Furthermore, the SUCRA analyses presented in Figure 5C indicated a high likelihood (61.53%) that PBL would be the most effective in boosting student satisfaction scores and subjective evaluations.

## Discussion

Recently, progress in computer technology and changes in the healthcare service system have made it challenging for medical students trained in traditional settings to fully adapt to the growing needs of public healthcare (28). Numerous innovative teaching methods have gained widespread adoption worldwide and shown promising results in improving teaching efficacy. However, there is uncertainty regarding the most effective teaching strategy for enhancing orthopedic teaching effectiveness due to the lack of direct comparative evidence. Compared to traditional pairwise meta-analysis, the NMA approach offers a more straightforward method and yields more insightful information (29). Consequently, we undertook this NMA to assess the impact of various teaching methods on student performance and satisfaction, considering theoretical exam scores, practical or clinical skill assessments, and student satisfaction ratings. By synthesizing all available evidence from 11 RCTs involving 690 medical students or residents in orthopaedics, our findings revealed that VR stands out as the most effective method for enhancing the practical or clinical skills of medical students or residents, whereas PBL has proven to offer a more effective overall learning experience throughout the educational journey.

The training of orthopedic interns has shifted from a primary focus on theoretical mastery to an emphasis on clinical application. Traditional LBL teaching tends to concentrate on imparting theoretical knowledge, which can hinder the development of students’ creativity and individual traits, and overlooks their subjective initiative and potential (30). Previous meta-analyses have found that PBL can improve knowledge scores compared to LBL. However, due to the relatively limited research on orthopedic education, no meta-analysis has been conducted to compare the effects of FC combined with TBL (FC + TBL) versus LBL on orthopedic teaching outcomes. While research indicates that both TBL and FC are more effective in enhancing student learning compared to traditional LBL, each method has its limitations (31, 32). Some students have provided feedback indicating that although the FC offers high-quality instructional resources, it demands strong self-learning abilities and offers limited opportunities for student interaction, potentially hindering the learning effectiveness of certain students (33, 34). Others have highlighted that TBL requires a substantial amount of time to master and necessitates a consistent schedule among team members (15). However, when these two approaches are integrated, they compensate for each other’s shortcomings, enabling students to engage with one another, acquire a broad range of knowledge in a brief period, and allocate more time to preparing for in-class problem discussions. More crucially, this combination fosters group discussions and critical thinking, enhances active communication abilities, and promotes a more relaxed atmosphere in the classroom (35). The apparent advantage of FC + TBL may arise from synergistic pedagogical mechanisms. The FC fosters self-directed cognitive preparation, while TBL promotes collaborative knowledge application through structured problem-solving. This combination aligns with constructivist theory—where learners actively construct knowledge through experience—and social interdependence theory, which posits that positive group interdependence enhances motivation, accountability, and deep learning. In our study, when assessing the impact of orthopedic teaching on exam scores, we also found that FC + TBL is the most effective teaching method, with an 81.73% likelihood of effectiveness. This is consistent with the findings of previous study (36).

The clinical skill test scores, which assess students’ competence in essential surgical techniques, offer a direct indication of the efficacy of hands-on training. Multiple systematic reviews have examined the advantages of incorporating VR technology into orthopaedic training programs. These reviews have consistently shown that VR notably improves both the theoretical understanding and practical abilities of learners, ranging from beginners to seasoned professionals like orthopaedists and neurosurgeons (37). Particularly, the integration of VR into surgical training for spinal interventions marks a novel approach to honing residents’ skills and enhancing their self-assurance (21). Our NMA revealed that VR significantly outperforms traditional LBL significantly (SMD = 6.88, 95% CI: 1.05 to 12.13), with a 62.07% probability of being the most effective method for improving procedural or clinical skill test scores. These results further highlight VR’s potential as a highly efficient and cost-effective training tool, effectively bridging the gap between theoretical knowledge and practical application.

A crucial metric for assessing teaching methods is student satisfaction. Prior systematic reviews have indicated that students in the PBL group exhibit greater interest in and satisfaction with teaching (38). Our analysis revealed that according to the SUCRA analysis, there is a 61.53% probability that PBL is the most effective approach for enhancing student satisfaction scores and subjective evaluations. PBL encourages students’ proactive participation, fosters their learning abilities, and boosts their enthusiasm for learning. Norman et al. (39) demonstrated that PBL enhances students’ learning interest, self-learning capabilities, and sustains these interests over time. Another study found that students in the PBL group outperformed those in the traditional teaching group in terms of professional knowledge and classroom satisfaction (*p* < 0.05) (22). Likewise, Ren et al. (40) reported that PBL significantly improved satisfaction among both students and teachers, aligning with our meta-analysis findings. Nevertheless, several pivotal factors, such as small group sizes and realistic case scenarios, influence teaching satisfaction (41).

This research consolidates and strengthens the existing evidence base in the academic literature on orthopedic education, advocating for innovative teaching approaches. To our understanding, previous systematic reviews and NMAs have assessed the impact of novel teaching methods on particular majors and curricula, including medicine (27), nursing (42), and pharmacology (43). However, our study represents the inaugural systematic review and NMA to explore the effects of innovative teaching strategies specifically in orthopaedics, thereby addressing a gap in orthopedic education. Orthopaedics differs from other surgical specialties (e.g., neurosurgery, general surgery) in several critical ways that shape educational needs: (1) it emphasizes hands-on psychomotor skills (e.g., fracture reduction, implant placement, arthroscopic manipulation) that require precise spatial reasoning and muscle memory—skills that may respond differently to instructional strategies than the cognitive or procedural focus of other fields; (2) orthopaedic training covers a broad spectrum of subspecialties (e.g., trauma, spine, sports medicine, joint replacement), each with distinct technical and decision-making demands; (3) orthopaedic trainees often balance high operative volume with didactic learning, creating unique time constraints that influence the applicability of teaching strategies (e.g., concise, simulation-based training may be more feasible than lengthy group discussions). Additionally, orthopaedic surgeons-in-training face distinct challenges, such as adapting to rapidly evolving implant technologies and navigating the physical demands of surgical procedures, which differ from the training priorities of other surgical specialists (e.g., neurosurgeons may focus more on imaging interpretation and microsurgical precision). By focusing exclusively on orthopaedics, our study provides targeted evidence for educators in this specialty, who previously lacked a synthesized overview of which innovative strategies align with their trainees’ unique needs. This specificity enhances the translatability of our findings to clinical practice, making it a core strength of the study. The NMA findings offer a ranked order of eight teaching strategies based on their effectiveness in mesh fixation, guiding curriculum designers and educators in adopting these novel instructional techniques. Furthermore, to ensure comprehensiveness and minimize selection bias, two authors independently conducted the study selection, data extraction, and quality assessment processes.

While FC + TBL and VR show promise, their real-world adoption requires careful consideration of feasibility. VR implementation entails significant costs for hardware, software licensing, and faculty training. FC + TBL demands curriculum redesign and skilled facilitation. We recommend phased integration—starting with pilot programs in well-resourced institutions—and the development of open-access TBL modules to support scalability in low-resource settings.

Despite its strengths, our study also has some limitations that should be acknowledged. Firstly, this NMA assesses only seven distinct novel teaching strategies, excluding other modes and various combinations of methodologies. Each teaching method incorporates a limited number of studies, with a maximum of five. The intricacy stemming from the amalgamation of diverse teaching approaches may impede the conduct of the NMA. Nonetheless, this NMA can aid in pinpointing effective teaching strategies and optimizing course arrangement. Secondly, the nature of the instructional approach precludes blinding of both students and instructors. Consequently, students may modify their behavior when aware of being studied under different teaching strategies, thereby affecting the reliability of evidence in RCTs. This limitation is an inherent and crucial shortcoming in all primary studies included in this meta-analysis. Given that pre-registration of protocols is not mandatory in educational research, the bias related to selective reporting of results does not apply when assessing literature quality, which impacts the quality evaluation of the included studies. Thirdly, this study is restricted to English-language publications, potentially overlooking trials with negative outcomes that may exist in extensive national databases, such as those in Chinese literature (44). Fourthly, differences in baseline characteristics among students, including residents and medical students in the included studies, the broad design framework, and variations in teacher expertise levels may introduce a certain degree of heterogeneity (45). Moreover, NMA differs from direct comparison and carries an additional risk of bias, particularly when few direct comparisons exist between the novel teaching strategies in our NMA, potentially leading to imprecision. Significant heterogeneity and imprecision in the data substantially degrade the quality of evidence, reducing the accuracy of results. A further limitation is the overrepresentation of Chinese orthopaedic residents, which may limit generalizability to Western or low-resource settings. Educational and cultural differences may affect engagement with strategies like TBL (46). Generational factors also matter: our predominantly Gen Z cohort tends to favor technology-enhanced tools like VR, which may not reflect preferences in other regions or age groups (47). Gender disparities in orthopaedics (e.g., lower female representation in some settings) could further influence participation in collaborative methods (48). Moreover, promising interventions such as VR and high-fidelity 3D simulations require resources often unavailable in low- and middle-income countries. Future research should test low-cost alternatives (e.g., free online modules, low-fidelity simulations) and include more diverse international samples to improve external validity. These contextual factors likely contributed to the modest effects observed for some innovative strategies, underscoring their context-dependent efficacy. In addition, our analysis included 11 RCTs involving 690 participants; however, many comparisons—such as VR for theoretical knowledge—were informed by a single study. This sparsity may reduce the precision and stability of effect estimates for certain nodes. Although sensitivity analyses excluding high-risk-of-bias studies yielded consistent rankings, future large-scale, head-to-head randomized trials are essential to validate these findings. Lastly, this study does not comprehensively capture other vital qualities and skills of orthopedic residents or students in the “outcomes” section, such as case analysis ability, social and communication skills, problem-solving and self-learning capabilities, and subjective enthusiasm. Future research should involve direct comparisons among novel teaching strategies to identify optimal educational approaches for orthopedic education.

## Conclusion

In conclusion, our NMA have provided valuable insights into the effectiveness of novel teaching strategies in orthopaedics education. Among the strategies evaluated, FC + TBL and VR emerged as the most effective methods for improving theoretical test scores and procedural or clinical skill scores, respectively. However, it is important to note that the differences among the teaching strategies were relatively minor, and more research is needed to fully understand their relative advantages and disadvantages. Future studies should focus on larger sample sizes, diverse student populations, and a wider range of outcome measures to provide a more comprehensive evaluation of teaching strategies in orthopaedics education. Overall, our findings have important implications for curriculum designers and educators seeking to optimize the learning experience for orthopaedics students or residents.

## Data Availability

The original contributions presented in the study are included in the article/Supplementary material, further inquiries can be directed to the corresponding author.

## References

[1] Vallée N Tronchot A Casy T Thomazeau H Jannin P Maximen J . Virtual reality-based simulation improves rotator cuff repair skill: a randomized transfer validity study. Orthop Traumatol Surg Res. (2024):104053. doi: 10.1016/j.otsr.2024.104053 39549736

[2] Wang L Xia Y Qiu C Yuan S Liu X. Comparative studies of the differences between flipped class and traditional class in orthopedic surgery education. Front Educ. (2024) 9:1382948. doi: 10.3389/feduc.2024.1382948

[3] Xue M Liu P Zhang J Sun Y Fang Y Yang J . Does a video-based and 3D animation hybrid learning system improve teaching outcomes in Orthopedic surgery? A randomized controlled trial. J Surg Educ. (2024) 81:1305–19. doi: 10.1016/j.jsurg.2024.05.015, 38944585

[4] Shuai L Huiwen W Shihao D Li J. The application of flipped classroom combined with team-based learning in the orthopedic clinical teaching. Medicine. (2023) 102:e35803. doi: 10.1097/MD.0000000000035803, 37904444 PMC10615534

[5] Back DA Von Malotky J Sostmann K Hube R Peters H Hoff E. Superior gain in knowledge by podcasts versus text-based learning in teaching orthopedics: a randomized controlled trial. J Surg Educ. (2017) 74:154–60. doi: 10.1016/j.jsurg.2016.07.008, 27651055

[6] Zhang W Xie Z Li J Liu C Wang Z Xie Y . Investigating the impact of virtual simulation experiment and massive open online course (MOOC) on medical students’ wound debridement training: a quasi-experimental study. BMC Med Educ. (2024) 24:1023. doi: 10.1186/s12909-024-05991-1, 39294595 PMC11409524

[7] Zhang W Li Z Li P Liu C Wang Z Liu Y . MOOC-virtual simulation integration enhances surgical clinical skills: a quasi-experimental study. Med Educ Online. (2025) 30:2579396. doi: 10.1080/10872981.2025.2579396, 41186279 PMC12587814

[8] Vernon DT Blake RL. Does problem-based learning work? A meta-analysis of evaluative research. Acad Med. (1993) 68:550. doi: 10.1097/00001888-199307000-00015, 8323649

[9] Zhang Y Zhou L Liu X Liu L Wu Y Zhao Z . The effectiveness of the problem-based learning teaching model for use in introductory Chinese undergraduate medical courses: a systematic review and meta-analysis. PLOS ONE. (2015) 10:e0120884. doi: 10.1371/journal.pone.0120884 25822653 PMC4378971

[10] Polyzois I Claffey N Mattheos N. Problem-based learning in academic health education. A systematic literature review. Eur J Dental Educ. (2010) 14:55–64. doi: 10.1111/j.1600-0579.2009.00593.x 20070800

[11] Li T Hu Y Song R Liu H Gao X Wang F . Unveiling the ultimate advantage: a meta-analysis of 3D visualization and problem-based learning in orthopedic education. BMC Med Educ. (2025) 25:60. doi: 10.1186/s12909-025-06654-5, 39806348 PMC11730799

[12] Ellaway RH Pusic M Yavner S Kalet AL. Context matters: emergent variability in an effectiveness trial of online teaching modules. Med Educ. (2014) 48:386–96. doi: 10.1111/medu.12389, 24606622

[13] Page MJ McKenzie JE Bossuyt PM Boutron I Hoffmann TC Mulrow CD . The PRISMA 2020 statement: an updated guideline for reporting systematic reviews. BMJ. (2021) 372:n71. doi: 10.1136/bmj.n71 33782057 PMC8005924

[14] Cornell JE. The PRISMA extension for network meta-analysis: bringing clarity and guidance to the reporting of systematic reviews incorporating network meta-analyses. Ann Intern Med. (2015) 162:797–8. doi: 10.7326/M15-0930, 26030637

[15] DeJongh B Lemoine N Buckley E Traynor L. Student preparation time for traditional lecture versus team-based learning in a pharmacotherapy course. Curr Pharm Teach Learn. (2018) 10:360–6. doi: 10.1016/j.cptl.2017.11.009, 29764641

[16] Sterne JAC Savović J Page MJ Elbers RG Blencowe NS Boutron I . RoB 2: a revised tool for assessing risk of bias in randomised trials. BMJ. (2019) 366:l4898. doi: 10.1136/bmj.l4898 31462531

[17] Cochrane (2024) Cochrane handbook for systematic reviews of interventions. Available online at: https://training.cochrane.org/handbook/current (Accessed February 14, 2025)

[18] Hu D O’Connor AM Wang C Sargeant JM Winder CB. How to conduct a Bayesian network meta-analysis. Front Vet Sci. (2020) 7:271. doi: 10.3389/fvets.2020.00271, 32509807 PMC7248597

[19] Higgins JPT Jackson D Barrett JK Lu G Ades AE White IR. Consistency and inconsistency in network meta-analysis: concepts and models for multi-arm studies. Res Synth Methods. (2012) 3:98–110. doi: 10.1002/jrsm.1044, 26062084 PMC4433772

[20] van Valkenhoef G Dias S Ades AE Welton NJ. Automated generation of node-splitting models for assessment of inconsistency in network meta-analysis. Res Synth Methods. (2016) 7:80–93. doi: 10.1002/jrsm.1167, 26461181 PMC5057346

[21] Capitani P Joil R Colonna C Schirò GR Legrenzi S Prandoni L . Virtual reality for surgical training in balloon kyphoplasty procedure. Eur J Orthop Surg Traumatol. (2024) 35:12. doi: 10.1007/s00590-024-04123-1, 39567395

[22] Sun M Chu F Gao C Yuan F. Application of the combination of three-dimensional visualization with a problem-based learning mode of teaching to spinal surgery teaching. BMC Med Educ. (2022) 22:840. doi: 10.1186/s12909-022-03931-5, 36471362 PMC9724438

[23] Lohre R Bois AJ Pollock JW Lapner P McIlquham K Athwal GS . Effectiveness of immersive virtual reality on Orthopedic surgical skills and knowledge acquisition among senior surgical residents: a randomized clinical trial. JAMA Netw Open. (2020) 3:e2031217. doi: 10.1001/jamanetworkopen.2020.31217, 33369660 PMC7770558

[24] Logishetty K Rudran B Cobb JP. Virtual reality training improves trainee performance in total hip arthroplasty: a randomized controlled trial. Bone Joint J. (2019) 101:1585–92. doi: 10.1302/0301-620X.101B12.BJJ-2019-0643.R1 31786991

[25] Hooper J Tsiridis E Feng JE Schwarzkopf R Waren D Long WJ . Virtual reality simulation facilitates resident training in Total hip arthroplasty: a randomized controlled trial. J Arthroplast. (2019) 34:2278–83. doi: 10.1016/j.arth.2019.04.002, 31056442

[26] Cong L Yan Q Sun C Zhu Y Tu G. Effect of problem and scripting-based learning on spine surgical trainees’ learning outcomes. Eur Spine J. (2017) 26:3068–74. doi: 10.1007/s00586-017-5135-2, 28526918

[27] Zhang S-L Ren S-J Zhu D-M Liu T-Y Wang L Zhao J-H . Which novel teaching strategy is most recommended in medical education? A systematic review and network meta-analysis. BMC Med Educ. (2024) 24:1342. doi: 10.1186/s12909-024-06291-4, 39574112 PMC11583476

[28] Frenk J Chen L Bhutta ZA Cohen J Crisp N Evans T . Health professionals for a new century: transforming education to strengthen health systems in an interdependent world. Lancet. (2010) 376:1923–58. doi: 10.1016/S0140-6736(10)61854-5, 21112623

[29] Watt J Tricco AC Straus S Veroniki AA Naglie G Drucker AM. Research techniques made simple: network meta-analysis. J Invest Dermatol. (2019) 139:4–12.e1. doi: 10.1016/j.jid.2018.10.028 30579427

[30] Zhao B Potter DD. Comparison of lecture-based learning vs discussion-based learning in undergraduate medical students. J Surg Educ. (2016) 73:250–7. doi: 10.1016/j.jsurg.2015.09.016, 26572094

[31] Ulfa Y Igarashi Y Takahata K Shishido E Horiuchi S. A comparison of team-based learning and lecture-based learning on clinical reasoning and classroom engagement: a cluster randomized controlled trial. BMC Med Educ. (2021) 21:444. doi: 10.1186/s12909-021-02881-8, 34419030 PMC8379851

[32] Salih KEMA El-Samani E-FZ Bilal JA Hamid EK Elfaki OA Idris MEA . Team-based learning and lecture-based learning: comparison of Sudanese medical students’ performance. Adv Med Educ Pract. (2021) 12:1513–9. doi: 10.2147/AMEP.S331296 34992488 PMC8713705

[33] Kuo YC Lin YH Wang TH Lin HCK Chen JI Huang YM. Student learning effect using flipped classroom with WPSA learning mode - an example of programming design course. Innov Educ Teach Int. (2023) 60:824–35. doi: 10.1080/14703297.2022.2086150

[34] Chang Y-H Lin J-Y Lu Y-T. Enhancing the intention to preview learning materials and participate in class in the flipped classroom context through the use of handouts and incentivisation with virtual currency. Sustainability. (2021) 13:3276. doi: 10.3390/su13063276

[35] Smeby SS Lillebo B Slørdahl TS Berntsen EM. Express team-based learning (eTBL): a time-efficient TBL approach in neuroradiology. Acad Radiol. (2020) 27:284–90. doi: 10.1016/j.acra.2019.04.022, 31186155

[36] Langdorf MI Anderson CL Navarro RE Strom S McCoy CE Youm J . Comparing the results of written testing for advanced cardiac life support teaching using team-based learning and the “flipped classroom” strategy. Cureus. (2018) 10:e2574. doi: 10.7759/cureus.2574, 30013860 PMC6039154

[37] Lohre R Wang JC Lewandrowski K-U Goel DP. Virtual reality in spinal endoscopy: a paradigm shift in education to support spine surgeons. J Spine Surg. (2020) 6:S208–23. doi: 10.21037/jss.2019.11.16, 32195429 PMC7063305

[38] Li T Song R Zhong W Liao W Hu J Liu X . Use of problem-based learning in orthopaedics education: a systematic review and meta-analysis of randomized controlled trials. BMC Med Educ. (2024) 24:253. doi: 10.1186/s12909-024-05244-1, 38459551 PMC10921736

[39] Norman GR Wenghofer E Klass D. Predicting doctor performance outcomes of curriculum interventions: problem-based learning and continuing competence. Med Educ. (2008) 42:794–9. doi: 10.1111/j.1365-2923.2008.03131.x, 18564299

[40] Ren X Yin J Wang B Roy Schwarz M. A descriptive analysis of medical education in China. Med Teach. (2008) 30:667–72. doi: 10.1080/01421590802155100, 18777425

[41] Kilgour JM Grundy L Monrouxe LV. A rapid review of the factors affecting healthcare students’ satisfaction with small-group, active learning methods. Teach Learn Med. (2016) 28:15–25. doi: 10.1080/10401334.2015.1107484, 26787081

[42] Ni J Wu P Huang X Zhang F You Z Chang Q . Effects of five teaching methods in clinical nursing teaching: a protocol for systematic review and network meta-analysis. PLoS One. (2022) 17:e0273693. doi: 10.1371/journal.pone.0273693, 36040919 PMC9426898

[43] Xiao C-L Ren H Chen H-Q Liu W-H Luo Z-Y Li W-R . Multidimensional evaluation of teaching strategies for pharmacology based on a comprehensive analysis involving 21,269 students. Front Pharmacol. (2023) 14:1145456. doi: 10.3389/fphar.2023.1145456, 37006996 PMC10050581

[44] Lin L Chu H. Quantifying publication bias in meta-analysis. Biometrics. (2018) 74:785–94. doi: 10.1111/biom.12817, 29141096 PMC5953768

[45] Burks TN. Improving student attitudes and academic performance in introductory biology using a project-based learning community. J Microbiol Biol Educ. (2022) 23:e00216-21. doi: 10.1128/jmbe.00216-21 35496694 PMC9053033

[46] Cai J Backer FD Vandermeersche G Lombaerts K. Comparing Chinese and Western classroom learning environment research: a bibliometric analysis and visualization. Front Psychol. (2023) 14:1213397. doi: 10.3389/fpsyg.2023.1213397, 37691787 PMC10484618

[47] Giordano V Bolonini J Fernandes F Pires RE Kojima K Labronici PJ . From baby boomers to gen Z: the changing values shaping medical education and professionalism in Brazil. Injury. (2025) 56:112836. doi: 10.1016/j.injury.2025.112836, 41135431

[48] Gilbert SR Torrez T Jardaly AH Templeton KJ Ode GE Coe K . A shadow of doubt: is there implicit bias among orthopaedic surgery faculty and residents regarding race and gender? Clin Orthop Relat Res. (2024) 482:1145. doi: 10.1097/CORR.0000000000002933, 38214651 PMC11219165

