# Supplementary material for: Identifying the most recommended novel teaching strategy in orthopaedics education: a systematic review and network meta-analysis
Source: Front Med (Lausanne). 2026 Feb 10;13:1762807. doi: 10.3389/fmed.2026.1762807 (PMC12929531; doi:10.3389/fmed.2026.1762807)
Supplement: Supplementary file 1 [file Supplementary_file_1.docx]

**Contents of supplemental appendix**

[Appendix 1. Full search strategy for each database 2](#_Toc190531594)

[Appendix 2. Risk of bias assessment for each included study 5](#_Toc190531595)

[Appendix 3. Results of pairwise meta-analyses for each outcome 6](#_Toc190531596)

[Appendix 4. Results of heterogeneity test for each outcome 7](#_Toc190531597)

# Appendix 1. Full search strategy for each database

**1. PubMed**

#1 “Students, medical”[MeSH Terms] OR “Medical students”[Title/Abstract] OR “Student, medical”[Title/Abstract] OR “Medical student”[Title/Abstract] OR “resident doctor” [Title/Abstract] OR “resident”[Title/Abstract]

#2 “Medical”[Title/Abstract] OR “Medicine”[Title/Abstract]) AND (“Student*”[Title/Abstract] OR “Undergraduate*”[Title/Abstract] OR “Graduate*”[Title/Abstract] OR “Pupil*”[Title/Abstract]

#3 #1 OR #2

#4 “orthopaedics surgery” [Title/Abstract] OR “Bone surgery operation” [Title/Abstract] OR “orthopedic surgery” [Title/Abstract] OR “orthopaedics” [Title/Abstract] OR “orthopedic” [Title/Abstract] OR “surgical operative” [Title/Abstract]

#5 #3 AND #4

#6 “Problem-Based Learning”[MeSH] OR “Problem Solving”[MeSH]

#7 “BOPPPS”[Title/Abstract] OR “Bridge-in, objective, pre-assessment, participatory learning, post-assessment, and summary”[Title/Abstract] OR “CBL”[Title/Abstract] OR “Case-based learning”[Title/Abstract] OR “PBL”[Title/Abstract] OR “Problem-based learning”[Title/Abstract] OR “TBL”[Title/Abstract] OR “Team-based learning”[Title/Abstract] OR “virtual reality” [Title/Abstract] OR “3D” [Title/Abstract] OR “Flipped classroom*”[Title/Abstract] OR “Simulation-Based”[Title/Abstract] OR “SBE” [Title/Abstract] OR “SBME”[Title/Abstract]

#8 #6 OR #7

#9 “Randomized Controlled Trial”[Publication Type] OR “Randomized Controlled

Trials as Topic”[Mesh]

#10 “Randomized controlled*”[Title/Abstract] OR (“Random*”[Title/Abstract] AND

(“control*”[Title/Abstract] OR “Compare*”[Title/Abstract]))

#11 #9 OR #10

#12 #5 AND #8 AND #11

**2. Web of science**

1: TS=(Students, medical) OR AB=(Medical students OR (Student, medical) OR Medical student) OR TS=(resident doctor OR resident)

2: TI=(Medical OR Medicine AND (Student* OR “Undergraduate* OR Graduate* OR “Pupil*)) OR TI=(resident doctor OR resident)

3: AB=(Medical OR Medicine AND (Student* OR “Undergraduate* OR Graduate* OR “Pupil*)) OR AB=(resident doctor OR resident)

4: #1 OR #2 OR #3

5: TI=(orthopaedics surgery) OR TI=(Bone surgery operation) OR TI=(orthopedic surgery) OR TI=(orthopaedics) OR TI=(orthopedic) OR TI=(surgical operative)

6: #4 AND #5

7: TS=(Problem-Based Learning OR Problem Solving)

8: TI=(BOPPPS OR (Bridge-in, objective, pre-assessment, participatory learning, post-assessment, and summary) OR CBL OR Case-based learning OR PBL OR Problem-based learning OR TBL OR Team-based learning OR virtual reality OR Flipped classroom* OR Simulation-Based OR Simulation OR SBE OR SBME)

9: AB=(BOPPPS OR (Bridge-in, objective, pre-assessment, participatory learning, post-assessment, and summary) OR CBL OR Case-based learning OR PBL OR Problem-based learning OR TBL OR Team-based learning OR virtual reality OR 3D OR Flipped classroom* OR Simulation-Based OR Simulation OR SBE OR SBME)

10: #7 OR #8 OR #9

11: TS=(Randomized Controlled Trials as Topic)

12: TI =(Randomized controlled* OR (Random* AND (control* OR Compare*)))

13: AB=(Randomized controlled* OR (Random* AND (control* OR Compare*)))

14: #11 OR #12 OR #13

15: #6 AND #10 AND #14

**3. The cochrane library**

#1 MeSH descriptor: [Students, Medical] explode all trees

#2 (Medical Students):ti,ab,kw OR (Medical Student):ti,ab,kw OR (Student, Medical):ti,ab,kw OR (resident doctor):ti,ab,kw OR (resident):ti,ab,kw

#3 #1 OR #2

#4 (Medical):ti,ab,kw OR (Medicine):ti,ab,kw OR (Medicalization*):ti,ab,kw

#5 (Student*):ti,ab,kw OR (Undergraduate*):ti,ab,kw OR (Graduate*):ti,ab,kw OR (Pupil*):ti,ab,kw

#6 #4 AND #5

#7 #3 OR #6

#8 (orthopaedics surgery):ti,ab,kw OR (Bone surgery operation):ti,ab,kw OR (orthopedic surgery):ti,ab,kw OR (orthopaedics):ti,ab,kw OR (orthopedic):ti,ab,kw OR (surgical operative):ti,ab,kw

#9 #7 AND #8

#10 MeSH descriptor: [Problem-Based Learning] explode all trees

#11 MeSH descriptor: [Problem Solving] explode all trees

#12 (Curriculum*, Problem-Based):ti,ab,kw OR (Problem-Based Curriculum*):ti,ab,kw OR (Learning, Problem-Based):ti,ab,kw OR (Problem-based learning):ti,ab,kw OR (PBL):ti,ab,kw (Medicine, Evidence-Based):ti,ab,kw OR (Medicine, Evidence Based):ti,ab,kw OR (Evidence Based Medicine):ti,ab,kw OR (BOPPPS):ti,ab,kw OR (Bridge-in, objective, pre-assessment, participatory learning, post-assessment, and summary):ti,ab,kw OR (CBL):ti,ab,kw OR (Case-based learning):ti,ab,kw OR (TBL):ti,ab,kw OR (Team-based learning):ti,ab,kw OR (virtual reality):ti,ab,kw OR (3D):ti,ab,kw OR (Flipped classroom*):ti,ab,kw OR (Simulation-Based):ti,ab,kw OR (Simulation):ti,ab,kw OR (SBE):ti,ab,kw OR (SBME):ti,ab,kw

#13 #10 OR #11 OR #12

#14 MeSH descriptor: [Randomized Controlled Trials as Topic] explode all trees

#15 (Randomized controlled*):ti,ab,kw OR ((Random*):ti,ab,kw AND ((control*):ti,ab,kw OR (Compar*):ti,ab,kw))

#16 #14 OR #15

#17 #9 AND #13 AND #16

**4. EMBASE**

#1 ‘medical student’/exp

#2 ‘Medical Student*’:ti,ab OR ‘Student*, Medical’:ti,ab OR ‘Resident*, Doctor’:ti,ab

#3 #1 OR #2

#4 ‘Medical’:ti,ab OR ‘Medicine’:ti,ab OR ‘Medicalization*’:ti,ab

#5 ‘Student*’:ti,ab OR ‘Undergraduate*’:ti,ab OR ‘Graduate*’:ti,ab OR ‘Pupil*’:ti,ab

#6 #4 AND #5

#7 #3 OR #6

#8 ‘orthopaedics surgery’:ti,ab OR ‘Bone surgery operation*’:ti,ab OR ‘orthopedic surgery’:ti,ab OR ‘orthopaedics*’:ti,ab OR ‘orthopedic’:ti,ab ‘surgical operative’:ti,ab

#9 #7 AND #8

#10 ‘Problem-Based Learning’/exp

#11 ‘Problem Solving’/exp

#12 ‘Curriculum*, Problem-Based’:ti,ab OR ‘Problem-Based Curriculum*’:ti,ab OR ‘Learning, Problem-Based’:ti,ab OR ‘Problem-based learning’:ti,ab OR ‘PBL’:ti,ab ‘Medicine, Evidence-Based’:ti,ab OR ‘Medicine, Evidence Based’:ti,ab OR ‘Evidence Based Medicine’:ti,ab OR ‘BOPPPS’:ti,ab OR ‘Bridge-in, objective, pre-assessment, participatory learning, post-assessment, and summary’:ti,ab OR ‘CBL’:ti,ab OR ‘Case-based learning’:ti,ab OR ‘TBL’:ti,ab OR ‘Team-based learning’:ti,ab OR ‘virtual reality’:ti,ab,kw OR ‘3D’:ti,ab,kw OR ‘Flipped classroom*’:ti,ab OR ‘Simulation-Based’:ti,ab OR ‘Simulation’:ti,ab OR ‘SBE’:ti,ab OR ‘SBME’:ti,ab

#13 #10 OR #11 OR #12

#14 ‘Randomized Controlled Trials as Topic’/exp

#15 ‘Randomized controlled*’:ti,ab OR ( ‘Random*’:ti,ab AND (‘control*’:ti,ab OR ‘Compare*’:ti,ab))

#16 #14 OR #15

#17 #9 AND #13 AND #16

# Appendix 2. Risk of bias assessment for each included study


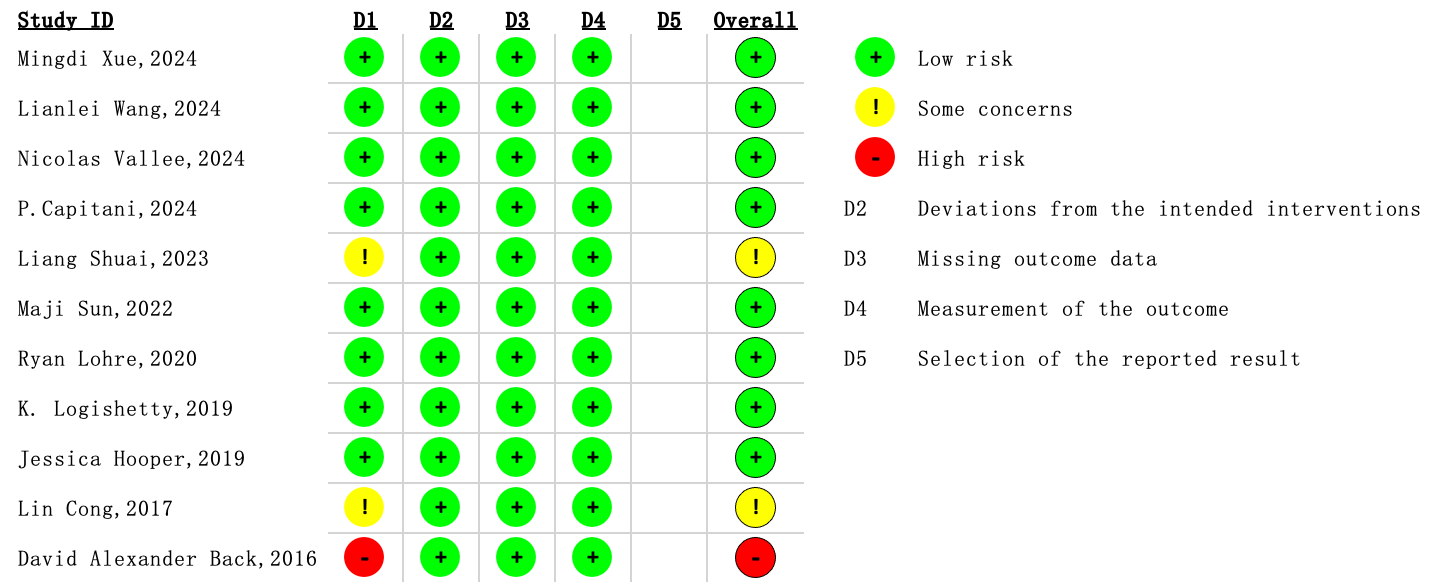


# Appendix 3. Results of pairwise meta-analyses for each outcome

**
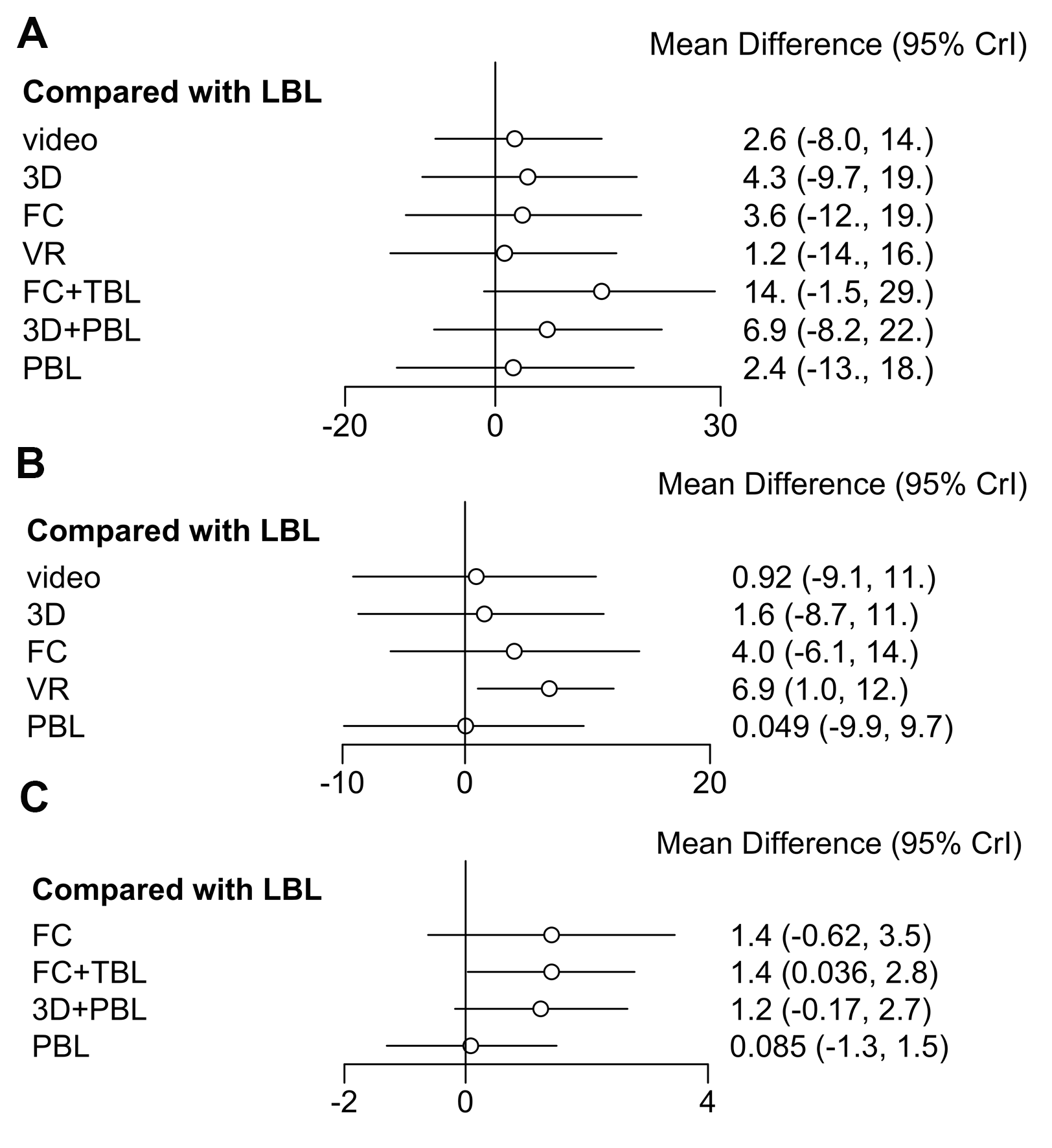
**

(A) Theoretical test scores; (B) Procedural or clinical skill scores; (C) Students’ satisfaction scores. PBL, problem-based learning; VR, virtual reality; 3D, three dimensions; FC, flipped classrooms; TBL, team-based learning; LBL, lecture-based learning; FC+TBL, flipped classrooms combined with team-based learning.

# Appendix 4. Results of Heterogeneity test for each outcome

**A. Theoretical test scores**


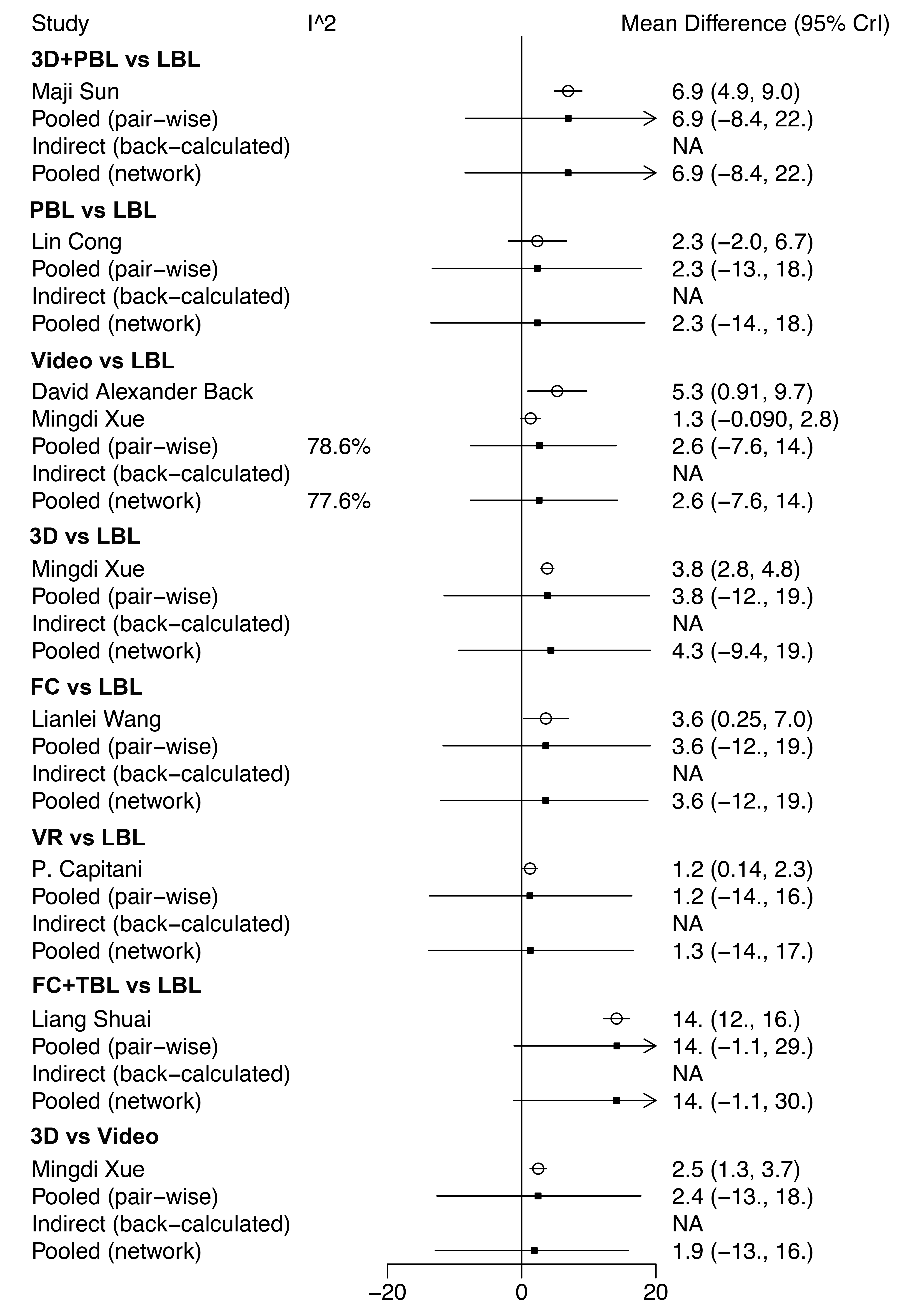


**B. Procedural or clinical skill scores**


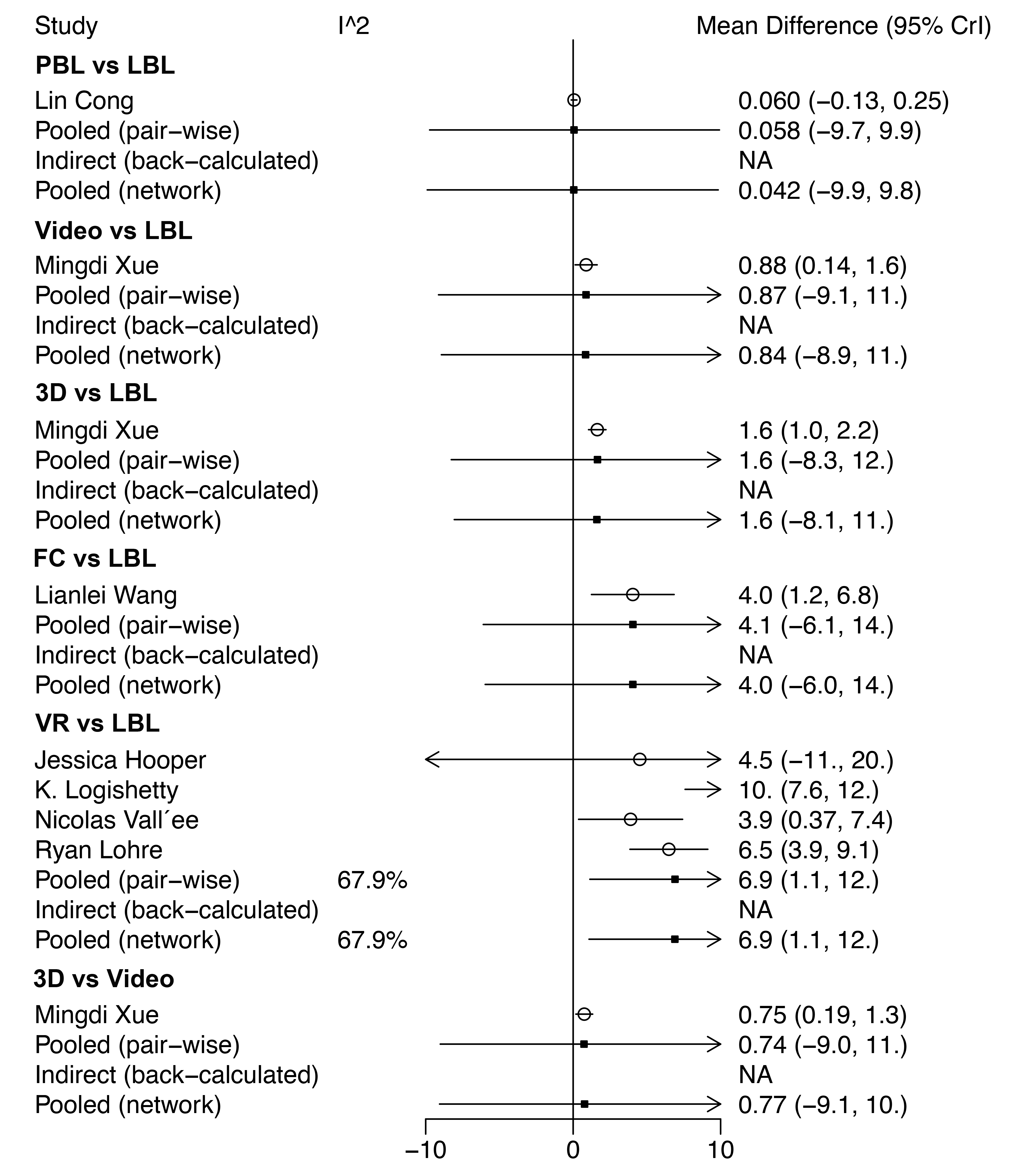


PBL, problem-based learning; VR, virtual reality; 3D, three dimensions; FC, flipped classrooms; TBL, team-based learning; LBL, lecture-based learning; FC+TBL, flipped classrooms combined with team-based learning.
